# Structure of the catalytic domain of the *Salmonella* virulence factor SseI

**DOI:** 10.1107/S0907444912039042

**Published:** 2012-11-09

**Authors:** Shyam S. Bhaskaran, C. Erec Stebbins

**Affiliations:** a Laboratory of Structural Microbiology, The Rockefeller University, New York, NY 10065, USA

**Keywords:** *Salmonella*, type III secretion, SPI-2, bacterial pathogenesis, cysteine proteases, SseI, SrfH

## Abstract

SseI is secreted into host cells by *Salmonella* and contributes to the establishment of systemic infections. The crystal structure of the C-terminal domain of SseI has been solved to 1.70 Å resolution, revealing it to be a member of the cysteine protease superfamily with a catalytic triad consisting of Cys178, His216 and Asp231 that is critical to its virulence activities. Structure-based analysis revealed that SseI is likely to possess either acyl hydrolase or acyltransferase activity, placing this virulence factor in the rapidly growing class of enzymes of this family utilized by bacterial pathogens inside eukaryotic cells.

## Abstract 2

The C-terminal domain of the *Salmonella* virulence factor SseI is structurally similar to the cysteine protease superfamily and contains the conserved catalytic triad characteristic of members of this family.

## 1. Introduction   

*Salmonella typhimurium* utilizes a highly specialized type 3 secretion system (T3SS) to deliver bacterial virulence factors from the microbial cytoplasm directly into host cells (Cornelis, 2006 ▶; Galán & Wolf-Watz, 2006 ▶). These injected proteins modulate host biochemical pathways to promote the replication of the pathogen (Abrahams & Hensel, 2006 ▶; Galán, 2009 ▶; Haraga *et al.*, 2008 ▶; Lilić & Stebbins, 2004 ▶; McGhie *et al.*, 2009 ▶; Srikanth *et al.*, 2011 ▶) and many have been shown to be mimics of host proteins in structure and biochemical function, although not necessarily at the sequence level (Schlumberger & Hardt, 2005 ▶; Stebbins & Galán, 2001 ▶).

*Salmonella* possesses two genomic ‘pathogenicity islands’ that encode separate but homologous T3SSs that are differentially activated depending on the location of the bacterium within the host (McGhie *et al.*, 2009 ▶; Rychlik *et al.*, 2009 ▶). *Salmonella* pathogenicity island 1 (SPI-1) is activated when any bacteria surviving the stomach reach the intestine and directs macropinocytosis of the bacteria into normally non­phagocytic intestinal epithelial cells (Schlumberger & Hardt, 2005 ▶). SPI-2 is activated following internalization and has been implicated in the creation and maintenance of the *Salmonella*-containing vacuole (SCV; Waterman & Holden, 2003 ▶) and the promotion of systemic infection within susceptible hosts (Ruby *et al.*, 2012 ▶).

SseI (also called SrfH) is an SPI-2-translocated protein that has been directly linked to systemic infection (McLaughlin *et al.*, 2009 ▶; Lawley *et al.*, 2006 ▶). Its N-terminal domain is required for translocation across the SCV and for subcellular targeting within the host. It contains an STE motif (∼1–140) that is common to all SPI-2 effectors encoded outside the SPI-­2 genetic locus and allows its localization and transport through T3SS-2 without the need for a cognate chaperone (Miao & Miller, 2000 ▶). Palmitoylation of Cys9 is then necessary for targeting of SseI to the plasma membrane once it is in the host cytoplasm (Hicks *et al.*, 2011 ▶). The N-terminal domain has also been shown to localize SseI to regions of high actin polymerization through an interaction with filamin (Miao *et al.*, 2003 ▶).

The C-terminal domain (∼140–322) of SseI contains its catalytic function (Hicks *et al.*, 2011 ▶) and shows some sequence similarity (19% identity over a 88-amino-acid stretch) to *Pasteurella multocida* toxin (PMT or ToxA), which causes atrophic rhinitis in swine (McLaughlin *et al.*, 2009 ▶). The structure of the C-terminal domain (1105–1285) of PMT is very similar to members of the cysteine protease superfamily and contains the active-site triad Cys1165, His1205, Asp1220. The enzymatic function of the PMT catalytic domain has recently been shown to be that of a glutamine deamidase, specifically modifying a Gln to a Glu in the α-subunit of heterotrimeric GTPases and leading to constitutive activation of their downstream signaling pathways (Kamitani *et al.*, 2011 ▶; Wilson & Ho, 2011 ▶). However, the enzymatic function of SseI remains unknown and functional studies have given conflicting results.

Yeast two-hybrid screens identified thyroid receptor-interacting protein 6 (TRIP6) as a possible interaction partner for SseI. TRIP6 is an adaptor protein that binds components of the Rac1 signaling pathway and is critical for cell motility and the NF-κB inflammatory pathways (Li *et al.*, 2005 ▶; Xu *et al.*, 2004 ▶). Transfection of macrophage-like cell lines (RAW276.7) confirmed the co-localization of TRIP6 and SseI (Worley *et al.*, 2006 ▶). It was also shown that RAW276.7 cells and JAWS dendritic cells migrated faster towards a chemoattractant when infected with wild-type (WT) rather than SseI-null strains. Furthermore, CD18+ macrophages infected with a WT strain were found in the bloodstream in significantly greater numbers than an SseI-mutant strain after oral infection. These results led the authors to postulate that SseI increases cellular migration, allowing infected macrophages to move actively into the bloodstream and to the other organs rather than passively through the lymphatic system, enhancing systemic infection (MacPherson *et al.*, 1995 ▶).

Immunoprecipitation with primary macrophage lysates identified IQGAP1 as another possible binding partner (McLaughlin *et al.*, 2009 ▶). IQGAP1 is a large scaffolding protein that binds actin and several small G proteins, playing a role in cellular motility and directionality (Brown & Sacks, 2006 ▶; Brown *et al.*, 2007 ▶). Transfection of bone-marrow-derived macrophages (BMDMs) with SseI showed its co-localization with IQGAP1 and polymerizing actin at the cell periphery. In contrast to earlier studies, an examination of cellular migration in BMDMs and dendritic cells revealed that SseI inhibited directed migration, leading to the hypothesis that inhibiting immune-cell migration in areas of infection allows the bacteria to evade clearance, leading to systemic infection. Further investigation revealed that the increased motility phenotype observed previously with RAW276.7 cells (Worley *et al.*, 2006 ▶) was potentially a consequence of increased detachment from the extracellular matrix in these types of cells rather than increased migration.

To address the questions around the biochemical function of SseI, we determined the crystal structure of its C-terminal domain to 1.7 Å resolution. The structure revealed that SseI is a member of the cysteine protease superfamily, containing a catalytic triad consisting of Cys178, His216 and Asp231. Structural similarity analysis places the enzymatic function of SseI in the family of acyl hydrolases or acyltransferases.

## 2. Materials and methods   

### 2.1. Cloning, expression and purification of SseI   

SseI (1–322) was cloned from *S. typhimurium* (strain LT2) genomic DNA into a modified pCDF-Duet (Novagen) vector containing two hexahistidine tags followed by a rhinovirus 3C protease cleavage site between the tag and the protein. Subsequent constructs based on secondary-structure prediction (Rost *et al.*, 2004 ▶) and limited proteolysis were subcloned from this original plasmid.

### 2.2. Protein purification   

All constructs were transformed into an *Escherichia coli* BL21 (DE3) strain and grown in LB medium at 310 K to stationary phase followed by induction with 0.1 m*M* IPTG at 293 K overnight. The cells were harvested by centrifugation, resuspended in 50 m*M* Tris pH 8.0, 10 m*M* imidazole, 200 m*M* NaCl, 1 m*M* PMSF and lysed using a homogenizer (Avestin). To digest DNA and decrease the viscosity, 0.1 mg ml^−1^ DNaseI and 5 m*M* MgCl_2_ were added to the lysate. After centrifugation, the lysates were loaded onto a gravity-flow column containing Ni–NTA resin (Qiagen) equilibrated in lysis buffer (without PMSF). This was followed by a wash (lysis buffer containing 30 m*M* imidazole) and protein elution (lysis buffer containing 250 m*M* imidazole). Following overnight dialysis into 50 m*M* Tris pH 8.0, 200 m*M* NaCl, 2 m*M* DTT and cleavage with 3C protease (1:100), the protein was run back over an Ni–NTA column to remove the tag. The flowthrough was then concentrated and further purified by gel-filtration chromatography on Superdex 75 and Superdex 200 columns (GE Healthcare) into 20 m*M* Tris pH 8.0, 200 m*M* NaCl, 2 m*M* DTT. Selenomethionine-substituted SseI (137–322) was produced by expression in the methionine-auxotrophic *E. coli* strain 834. Cells were grown for 6 h at 310 K in minimal medium with methionine replaced by seleno­methionine. After induction, the protein was purified as before except that DTT was used at 10 m*M* rather than 2 m*M* in all buffers to prevent Se oxidation.

### 2.3. Limited proteolysis   

Purified protein at ∼10 mg ml^−1^ (with added 5 m*M* CaCl_2_) was incubated for 30 min at room temperature and 277 K with increasing ratios (1:10 to 1:1000) of the nonspecific protease subtilisin (Sigma). The reactions were stopped by the addition of SDS-containing buffer and heating at 368 K for 10 min. Samples were run out on SDS–PAGE gels and transferred onto a PVDF membrane in the presence of 10 m*M* CAPS, 10% methanol pH 11.0 transfer buffer at 60 mV overnight. Protein bands were visualized by Ponceau (Sigma) staining and bands corresponding to stable fragments that persisted during proteolysis were sent to the Proteomics Resource Center at The Rockefeller University for Edman N-terminal sequencing.

### 2.4. Reductive methylation   

Purified SseI (137–322) was dialyzed into 50 m*M* HEPES pH 7.6, 150 m*M* NaCl and diluted to 0.4 mg ml^−1^ (300 ml). 40 µl 1 *M* formaldehyde (methanol-free) and 20 µl 1 *M* dimethyl­amine–borane complex (DMBA; Aldrich) were slowly added per millilitre of protein. After shaking for 2 h in the dark at 277 K, the previous step was repeated. This was followed by the addition of a further 10 µl DMBA per millilitre of protein and shaking overnight for 18 h. The reaction was quenched by the addition of 25 ml 1 *M* ammonium sulfate, after which the protein was concentrated and exchanged into 20 m*M* Tris pH 8.0, 2 m*M* DTT on an Superdex 75 column (GE Healthcare). Samples sent for total mass MALDI-TOF analysis at the W. M. Keck Foundation Biotechnology Resource Laboratory at Yale University indicated that 12 out of 13 possible sites (12 Lys and the N-terminus) were methylated.

### 2.5. Crystallization   

Crystallization screens were conducted using 1:1 sitting drops at room temperature and 277 K. The C-terminal domain (137–322; ∼20 mg ml^−1^) gave large rectangular crystals in 1.15 *M* LiSO_4_, 0.1 *M* sodium acetate pH 4.6 at 277 K when optimized (1:1 hanging drops). However, these crystals were very fragile and consisted of multiple lattices overlaid on each other as observed in their diffraction patterns. SseI (137–322) was then reductively methylated and rescreened. It gave multiple crystals of differing morphologies in a number of conditions containing MES, Tris and HEPES buffers pH 6.0–9.0 and 1.6–2.0 *M* ammonium sulfate. To obtain single crystals, crystals from 0.1 *M* MES pH 7.1, 1.8 *M* ammonium sulfate were crushed and microseeded into pre-equilibrated (2 h) drops containing protein/reservoir solution (0.1 *M* MES pH 5.5, 1.6 *M* ammonium sulfate). This procedure produced single small rectangular crystals. To obtain larger crystals, single crystals obtained in the previous step were macroseeded into pre-equilibrated drops, giving large thin rectangular plates that diffracted well. SeMet-substituted crystals of SseI (137–322) were obtained under the same conditions but gave large crystals directly without any seeding. The crystals were then cryoprotected by transfer into mother liquor supplemented with 25% glycerol in small increments.

### 2.6. Structure determination   

Data were collected on beamlines X3A and X29A at the Brookhaven National Synchrotron Light Source (NSLS) and the structure was solved by single-wavelength anomalous dispersion (SAD) to 1.7 Å resolution using the selenium peak (0.9790 Å) of the selenomethionine-substituted crystals. The crystals belonged to space group *P*4_1_2_1_2 (unit-cell parameters *a* = *b* = 52.26, *c* = 132.15 Å), with one molecule in each asymmetric unit. The data were processed using *HKL*-2000 (Otwinowski & Minor, 1997 ▶) and SAD phases were determined by using the *AutoSolve* workflow in the *PHENIX* suite involving multiple programs such as *HySS*, *Phaser* *etc.* (Adams *et al.*, 2010 ▶). A preliminary model was built into the resulting electron density by *ARP*/*wARP* (Perrakis *et al.*, 1999 ▶) and was then completed by repeated cycles of model building in *Coot* (Emsley & Cowtan, 2004 ▶) followed by refinement in *REFMAC* (Murshudov *et al.*, 2011 ▶). The native crystals only diffracted to 2.05 Å resolution and belonged to space group *P*2_1_2_1_2_1_ (unit-cell parameters *a* = 54.05, *b* = 63.19, *c* = 110.62 Å, α = β = γ = 90°). They also had a significantly higher crystal mosaicity (0.95° *versus* 0.50°). Data were processed using *HKL*-2000 (Otwinowski & Minor, 1997 ▶) and were phased by molecular replacement in *Phaser* (McCoy *et al.*, 2005 ▶) using the SeMet-derivative structure as a starting model. The solved model contained two molecules of the protein per asymmetric unit and was further built in *Coot* (Emsley & Cowtan, 2004 ▶) and refined in *REFMAC*5 (Murshudov *et al.*, 2011 ▶). All figures were generated using *PyMOL* (http://www.pymol.org). Multiple programs from the *CCP*4 suite were used at various stages (Winn *et al.*, 2011 ▶).

### 2.7. *In vitro* activity assays   

*In vitro* assays with small-molecule, peptide or generic protein (casein) substrates were conducted to test full-length SseI (1–322) and the C-terminal catalytic domain (137–322) for glutamine deamidase (Sigma), transglutaminase (Sigma), protease (EnzCheck Kit, Invitrogen) and *N*-acetyltransferase (Brooke *et al.*, 2003 ▶) activities. See Supplementary Material^1^ for details.

## 3. Results and discussion   

### 3.1. Domain determination   

Limited proteolysis (Koth *et al.*, 2003 ▶) of constructs 1–322, 1–138 and 137–322 and N-terminal Edman sequencing of the stable fragments indicated that SseI contains three distinct domains: a possible secretion signal (1–24), the STE translocation domain (24–130) and a catalytic domain (137–322) (Supplementary Fig. S1^1^).

### 3.2. Overall structure   

**Figure 1:**
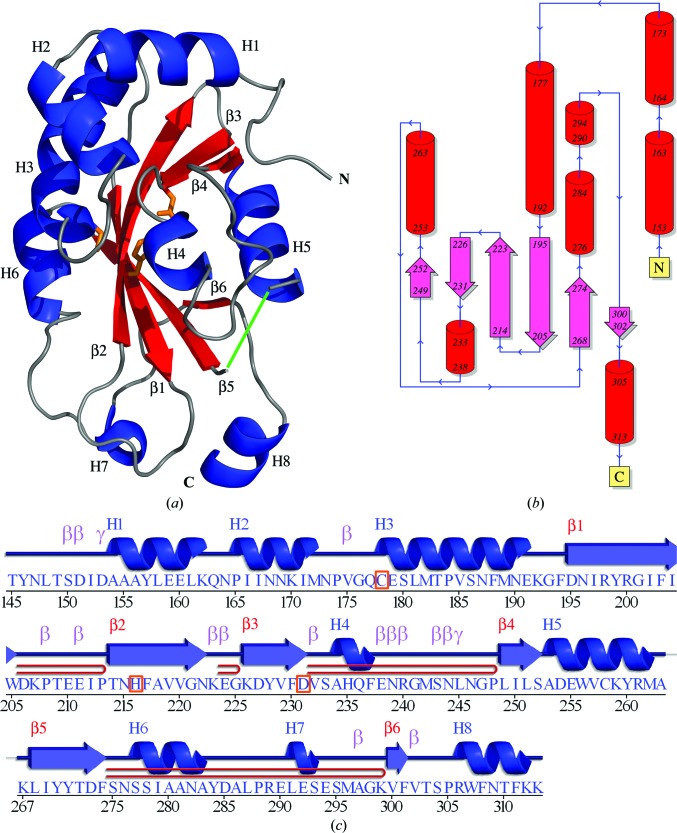
Overall structure of the catalytic domain: SseI (137–322). (*a*) Overall fold shown as a cartoon diagram. Each of the eight helices (blue) and six β-strands (red) are numbered, with the catalytic triad Cys178, His216 and Asp231 shown as sticks in orange. The missing residues 264–266 are shown as a green line. (*b*) Topology of the catalytic domain. (*c*) Secondary-structure alignment with the sequence of the catalytic domain generated by *PDBSum* (Laskowski, 2009 ▶). Helices are numbered with an H and strands with a β. β-­Turns are indicated by β, γ-turns by γ and β-hairpins by 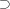.

Crystals of reductively methylated (Rayment, 1997 ▶) SeMet-derivative and native forms of SseI (37–322) were grown as described and their structures were solved using SAD (Adams *et al.*, 2010 ▶) and molecular replacement (McCoy *et al.*, 2005 ▶), respectively (Table 1 ▶). The overall structure and topology of the high-resolution (1.7 Å) SeMet-derivative structure (Fig. 1 ▶) revealed a single catalytic domain consisting of a core of six antiparallel β-sheets and eight surrounding helices. No density was observed for 12 residues at the N-terminus (137–144 and four vector-encoded residues), nine residues at the C-­terminus (314–322) or for residues 264–266 (a presumed disordered loop). The final model contained 166 amino acids spanning residues 145–313 and 137 waters, with *R* and *R*_free_ values of 18.3% and 21.4%, respectively.

The native crystals diffracted to 2.05 Å resolution and belonged to a different space group (*P*2_1_2_1_2_1_) to the SeMet-derivative crystals (*P*4_1_2_1_2). The native structure was solved using the SeMet-derivative structure as a starting model and showed two molecules of the protein per asymmetric unit. No density was observed for the first 12 and the last nine residues of each molecule. The final model contained two chains of 168 or 169 amino acids, spanning residues 145–313, and 158 waters, with *R* and *R*_free_ values of 19.4% and 23.8%, respectively (residue 177 of chain *B* was omitted owing to weak electron density). The dimer is linked by a disulfide bond between Cys258 of each chain on the opposite face of the molecule to the catalytic Cys176 (Supplementary Fig. S2^1^). The dimer is likely to be a crystal artifact since the C-terminal domain purifies as a monomer on size-exclusion chromatography. The SeMet protein was purified using 10 m*M* DTT instead of 2 m*M* DTT to prevent Se oxidation, which may explain why it did not crystallize as a disulfide-linked dimer under these reducing conditions. It might also be the reason why seeding was not required to obtain large diffracting crystals of the SeMet protein. The chain backbone in the native structure did not show any large deviations from the SeMet-derivative structure, with r.m.s.d.s of 0.277 Å (chain *A*) and 0.250 Å (chain *B*) between the two models. The higher resolution SeMet-derivative structure was used for all subsequent structural analyses.

### 3.3. Active site   

**Figure 2:**
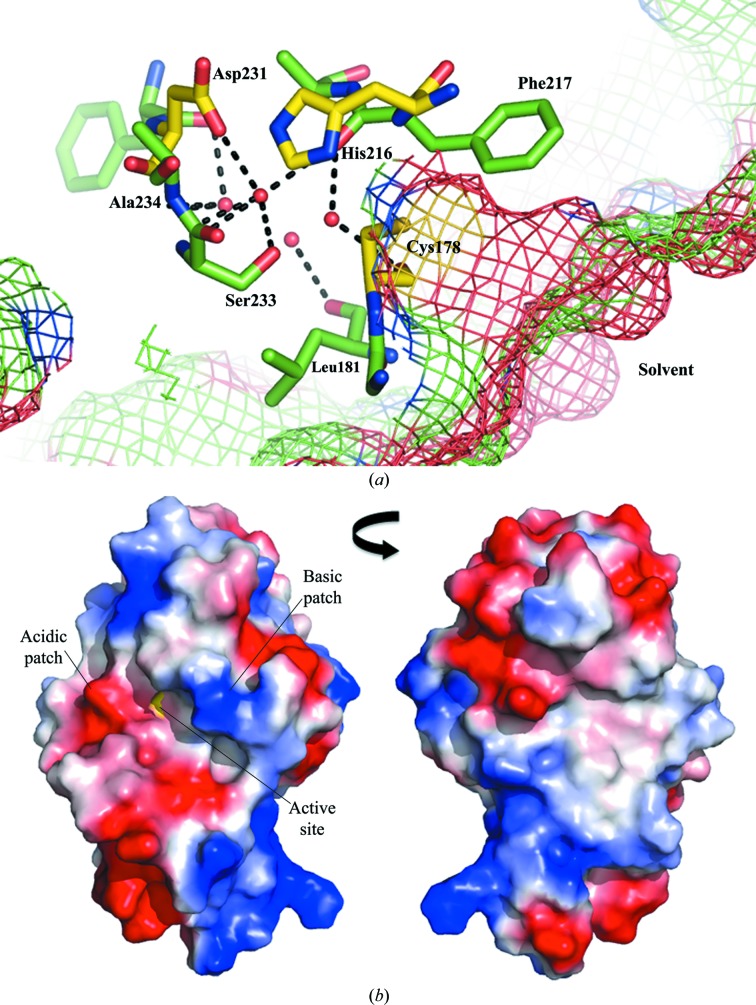
Active-site water molecules and surface electrostatics. (*a*) Residues making contact with the four waters present in the active-site cavity. The catalytic residues are in yellow, noncatalytic residues are in green and the mesh shows the surface of the molecule. (*b*) Electrostatic surface potentials of the catalytic domain. Two views rotated by 180° are shown, with the position of the active-site Cys178 in yellow.

The SseI structure shows significant similarity to those of members of the cysteine protease superfamily, which allowed the identification of the catalytic triad: Cys178, His216 and Asp231. Behind Cys178, there is a small cavity that contains four water molecules (Fig. 2 ▶ *a*). They form hydrogen bonds to the backbones of several residues that surround them, Leu181, Cys178, Phe217, Ser233 and Ala234, as well as an additional hydrogen bond to the side chain of Asp231. There is no room in this cavity for additional molecules and there are no other cavities from the surface that lead towards the active-site residues, making it likely that a small molecule such as water is involved in the catalytic mechanism of SseI.

The electrostatic surface potential map of the catalytic domain (Fig. 2 ▶ *b*) shows that there are multiple acidic and basic patches distributed around the molecule that could form binding surfaces for regulatory or substrate proteins. Looking at the region surrounding the active site, it is not obvious what type of substrate would be likely to bind, as there is both a positively charged and a negatively charged region on opposite sides of the solvent-accessible Cys178.

### 3.4. Structurally similar proteins   

To further investigate the enzymatic function of SseI, the catalytic domain structure was submitted to the *DALI* server (Holm *et al.*, 2008 ▶). All of the structural matches (Supplementary Figs. S3 and S4^1^) were from members of clan CA of the cysteine protease superfamily (EC 3.4.22), which is a large family spread across prokaryotes, eukaryotes and viruses. This family encodes a remarkably diverse set of enzymes possessing acyl-hydrolysis or acyl-transfer functions, including endo/exopeptidases, proteases, synthetases, deamidases, deubiquitinases, trans­glutaminases and acetyltransferases (Barrett & Rawlings, 2001 ▶; Rawlings *et al.*, 2009 ▶).

The active-site His deprotonates Cys, which then acts as a nucleophile attacking acyl (C=O) bonds at the C atom. The acyl bond is reformed by displacement of the Cys by water (in the case of hydrolysis) or another nucleophile (in the case of acyl transfer). A third residue (Asn/Gln/Asp/Glu) acts to stabilize the His during Cys deprotonation. These enzymes all have a common fold consisting of a core of antiparallel β-­sheets that is surrounded by helices, with the active-site cysteine perched at the end of one helix. Interestingly, all of the closest structural homologs to SseI are members of this family that possess a Cys-His-Asp/Glu catalytic triad, as opposed to the classical cysteine proteases such as papain, which have Cys-His-Asn catalytic triads.

**Figure 3:**
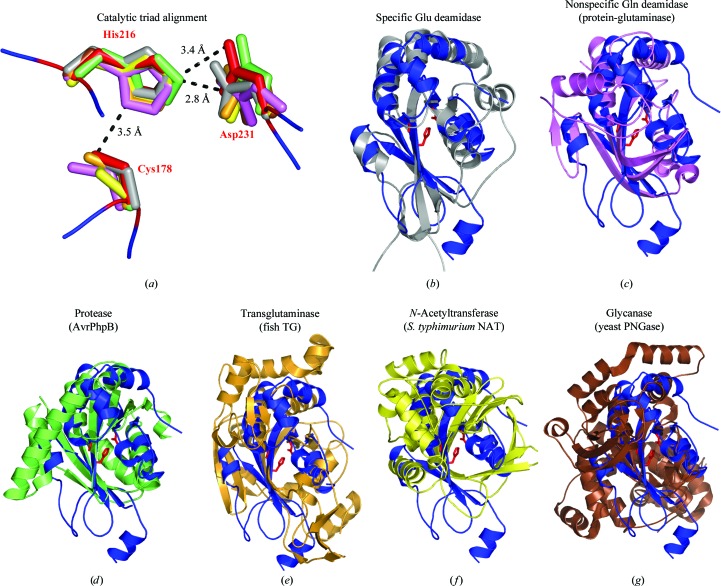
Structural similarity to the cysteine protease superfamily. (*a*) Alignment of the catalytic triads from representative structural matches to SseI. Cys178, His216 and Asp231 of SseI are shown in red and the distances between these residues are given in Å. (*b*–*e*) Structural alignments of SseI (blue; catalytic triad in red) with (*b*) PMT (gray; PDB entry 2ec5; residues 1105–1285; triad Cys1165/His1205/Asp1220; Kitadokoro *et al.*, 2007 ▶), (*c*) protein-glutaminase (magenta; PDB entry 2zk9; residues 1–185; triad Cys42/His83/Asp103; Hashizume *et al.*, 2011 ▶), (*d*) AvrPphB (green; PDB entry 1ukf; residues 81–268; triad Cys98/His212/Asp227; Zhu *et al.*, 2004 ▶), (*e*) sea bream transglutaminase (orange; PDB entry 1g0d; residues 141–460; triad Cys272/His332/Asp355; Noguchi *et al.*, 2001 ▶), (*f*) *S. typhimurium* NAT (yellow; PDB entry 1e2t; residues 1–271; triad Cys69/His107/Asp122; Sinclair *et al.*, 2000 ▶) and (*g*) yeast PNGase (brown; PDB entry 3esw; residues 1–329; triad Cys191/His218/Asp235; Zhao *et al.*, 2009 ▶).

The structural homologs of known function can be categorized into five families: Gln deamidases, transglutaminases (TGs), proteases, arylamine *N*-acetyl transferases (NATs) and peptide *N*-glycanases (PNGases). All of these activities rely on either the hydrolysis or the formation of an amide moiety. An alignment of the catalytic triads from representative members of each group is shown in Fig. 3 ▶(*a*). The measured Cys-to-His (3.5 Å) and His-to-Asp (2.8 and 3.4 Å) bond distances fit well to those expected for this type of catalytic triad.

Gln deamidases catalyze the conversion of glutamine residues to glutamate using water as the attacking moiety during hydrolysis. A substrate-specific de­amidase such as PMT only targets a particular Gln residue on its protein substrates, the Gα subunits of heterotrimeric G_q_/G_i_/G_12/13_ GTPases, leading to specific downstream effects through constitutive signaling of host mitogenic, calcium and cytoskeletal pathways (Orth & Aktories, 2012 ▶; Wilson & Ho, 2011 ▶). Fig. 3 ▶(*b*) shows the remarkable structural similarity of PMT and SseI (based on a *Z*-score of 7.5; Kitadokoro *et al.*, 2007 ▶). PMT is also the only structural homolog to show significant sequence similarity to SseI (20% over the 126-amino-acid structural alignment), also providing further evidence for a similar biochemical function.

Unlike PMT, nonspecific Gln deamidases act on any surface-exposed glutamines in both peptides and proteins. Fig. 3 ▶(*c*) shows an alignment with protein-glutaminase from *Chryseobacterium proteolyticum* (*Z*-score of 6.5; Yamaguchi *et al.*, 2001 ▶). The cores fit well, but protein-glutaminase contains two additional β-strands on the outside of the core that are not present in SseI.

Transglutaminases are a widespread group of Ca^2+^-dependent enzymes that catalyze the cross-linking of Gln and Lys residues between proteins or of Gln and free amines in the cell cytosol and extracellular matrix. They function as a molecular glue, polymerizing proteins and stabilizing them in tissues, and have been implicated in wound healing, apoptosis, angiogenesis and cellular regeneration (Griffin *et al.*, 2002 ▶). A structural alignment of SseI and the cysteine protease domain of red sea bream (a fish) TG is shown in Fig. 3 ▶(*e*) (*Z*-score of 4.6). TG has three domains in addition to its catalytic domain, which bind Ca^2+^ and have regulatory functions on activity, as well as separate binding sites for the acyl donor and the Gln acceptor (Noguchi *et al.*, 2001 ▶).

Cysteine proteases catalyze the hydrolysis of main-chain amide bonds. Fig. 3 ▶(*d*) shows an alignment with AvrPphB, a TTSS effector from the plant pathogen *Pseudomonas syringae* (*Z*-score 5.1; Zhu *et al.*, 2004 ▶). AvrPphB undergoes auto­processing (removing the first 62 amino acids) before becoming active and specifically cleaves the serine/threonine kinase PBS1. It is part of the YopT family of TTSS-trans­located cysteine proteases, all of which are processed in the same way (Shao *et al.*, 2002 ▶). However, there is no evidence of auto­processing in SseI. The cores fit well except for an extended helix protruding out from AvrPphB that is not present in SseI.

NATs catalyze the transfer of an acetyl group from acetyl-CoA to the terminal N atom of a wide range of hydrazine and arylamine compounds (Upton *et al.*, 2001 ▶). In prokaryotes they are usually involved in inactivating antibiotics (first identified in isoniazid-resistant *Mycobacterium tuberculosis*), while in mammals they play a role in drug metabolism and foreign-substance carcinogenesis in the liver. Fig. 3 ▶(*f*) shows an alignment with the cysteine protease domain of NAT from *S. typhimurium* (*Z*-score of 6.0; Sinclair *et al.*, 2000 ▶). The cores of the two cysteine protease domains match very well, but NAT contains multiple helical insertions, creating an extra lobe in the protein that is not present in SseI.

Peptide *N*-glycanase is involved in the deglycosylation of misfolded glycoproteins before they can be degraded by the proteosome and is widely conserved in eukaryotes. It specifically cleaves the bond between the Asn residue and the first GlcNAc residue of the glycan chain in N-linked glycoproteins (Suzuki, 2005 ▶). A structural alignment of SseI and the cysteine protease domain of yeast PNGase is shown in Fig. 3 ▶(*g*) (*Z*-­score of 4.8; Lee *et al.*, 2005 ▶). The cores of the two proteins match, but a distinguishing feature of PNGase is a deep binding cleft formed between the catalytic domain and an adjacent domain, along which the unfolded glycoprotein can bind. Such a binding cleft is missing from SseI, making it unlikely that it acts as a PNGase.

### 3.5. Possible enzymatic functions of the catalytic domain   

Based on the high structural similarity to the catalytic domain of PMT (*Z*-score of 7.5), their common evolutionary lineage (PMT is the only structural homolog that also shows sequence similarity to SseI) and the small active-site cavity present around the catalytic triad (consistent with a small molecule such as water being the attacking nucleophile during catalysis), we hypothesized that SseI was also a substrate-specific Gln deamidase like PMT.

Since target(s) of SseI have not yet been identified, we were limited to using a nonspecific *in vitro* Gln deamidase assay to check for deamidation activity (measuring ammonia release from the peptide substrate Z-Gln-Gly or from methylated casein; Supplementary Fig. S5^1^). Both full-length SseI (1–322) and the catalytic domain (137–322) were tested since it is possible that the N-terminal STE domain could be binding to the catalytic domain and inhibiting its activity when not in the presence of its correct binding partners/substrates, as has been shown in the case of the TTSS-2 effector SspH2 (Quezada *et al.*, 2009 ▶). Moreover, the separate PMT catalytic domain has been shown to be more active than the full-length toxin when tested in *in vitro* assays with recombinant Gα subunits (Kamitani *et al.*, 2011 ▶). However, no Gln deamidase activity was observed with either substrate. Further *in vitro* assays for transglutaminase, protease and *N*-acetyltransferase functions also did not show any enzyme activity (Supplementary Figs. S6–S8^1^).

The lack of activity in the biochemical assays with generic substrates does not necessarily mean that SseI does not have Gln deamidase activity (or possibly one of the other tested activities). For instance, there is no evidence that PMT deamidates any other glutamines in its targets other than a single conserved Gln, with mass spectrometry showing only a single Da shift at Gln205 of Gα_i_ when it is co-expressed with PMT (Orth *et al.*, 2009 ▶). Similarly, SseI might also exhibit a high substrate specificity, and definitive assays to determine its enzymatic function might only be possible once its host target(s) have been identified.

If SseI behaves like PMT, targeting GTPase signaling by deamidating critical Gln residues involved in GTP hydrolysis and converting the GTPase to a constitutively ‘on’ form, the list of possible substrates is extensive and includes the membrane-bound heterotrimeric GTPase superfamily (including the G_i_, G_s_, G_q_ and G_12/13_ subfamilies; Neves *et al.*, 2002 ▶; McCudden *et al.*, 2005 ▶; Bhattacharya *et al.*, 2004 ▶), as well as the cytosolic or membrane-localized members of the Ras GTPase superfamily (Ras, Rho, Rab, Ran and Arf subfamilies; Rojas *et al.*, 2012 ▶; Colicelli, 2004 ▶). Based on the localization of SseI to regions of active actin polymerization and its involve­ment in cell motility and adhesion, the Rho GTPase subfamily are good candidates as SseI substrates. In particular, Cdc42, Rac1 and RhoA all have direct effects on cellular movement, cytoskeletal rearrangement, cellular adhesion and cell polarity. Moreover, these Rhos are also targeted by other bacterial toxins (Lemonnier *et al.*, 2007 ▶). For instance, all three are deamidated by CNF1 (cytotoxic necrotizing factor) from *E. coli* and are transglutaminated with small cytosolic amines by DNT (dermonecrotic toxin) from *Bordetella*, specifically at Gln61 (Cdc42/Rac1) or Gln63 (RhoA).

Another variable that could complicate the identification of the biochemical function of SseI (even with the correct substrate) is the requirement for additional bacterial or host factors for the activation of cysteine protease domains that has been observed in some virulence effectors. For instance, the *Vibrio cholerae* toxin RTX (a member of the multifunctional MARTX family of toxins) has a catalytic domain that is activated by inositol hexakisphosphate (InsP_6_) once it is has been transported into the host cytosol. InsP_6_ binding leads to toxin autoproteolysis by the catalytic domain and the release of its other effector domains. The structure of the InsP_6_–RTX complex showed that the InsP_6_ allosteric binding site was distinct from the catalytic site, indicating that it was not acting as a cofactor (Lupardus *et al.*, 2008 ▶). Three such possibilities for such an activating role with SseI are filamin and TRIP6, which were identified as possible interaction partners through yeast two-hybrid screens (although not confirmed biochemically; Miao *et al.*, 2003 ▶; Worley *et al.*, 2006 ▶), and IQGAP1, for which a direct interaction with SseI has been shown both in macrophages and *in vitro* (McLaughlin *et al.*, 2009 ▶).

## 4. Conclusions   

In this work, we were able to determine the domain organization of SseI and obtain a crystal structure of its catalytic C-­terminal domain (reductively methylated; residues 137–322) by SAD to 1.70 Å resolution. Based on its structural matches, SseI is a member of a particular subgroup (clan CA) of the cysteine protease superfamily. These enzymes have acyl hydrolase and acyltransferase activities towards a wide range of amide-derived moieties. Based on the structural alignments, the catalytic triad of SseI was identified as consisting of Cys178, His216 and Asp231. Furthermore, the structural matches could be categorized into five biochemical activities: Gln deamidase, transglutaminase, protease, *N*-acetyltransferase and peptide *N*-glycanase.

*In vitro* assays based on generic small-molecule, peptide and protein substrates were performed for the activities described (except for peptide *N*-glycanase, since the structural requirements for the binding of its glycosylated substrate are not present in SseI), but neither the full-length enzyme nor the isolated catalytic domain showed any enzymatic activity in the assays. However, this lack of activity does not rule out these enzymatic functions, as SseI could have a very strong substrate specificity. For instance, its closest structural (and sequence) match, PMT, has deamidase activity towards a single specific conserved Gln in the Gα subunit of heterotrimeric G proteins (the conversion of Gln to Glu disrupts the intrinsic GTPase activity of these enzymes, keeping them constitutively active), but does not deamidate any other Gln residues in its targets.

If SseI behaves similarly to PMT and functions by modulating cellular signaling downstream of host GTPases, its possible substrates are extensive and include the heterotrimeric GTPase superfamily and the Ras GTPase superfamily. The members of the Rho GTPase subfamily (part of the Ras superfamily; Rho, Rac and Cdc42) present intriguing candidates for SseI substrates for several reasons: (i) the cellular localization of SseI and the observed phenotypes of mutant bacteria lacking this virulence factor; (ii) the involve­ment of these small G proteins in cellular movement, cytoskeletal rearrangement, cellular adhesion and cell polarity; and (iii) the common targeting of this family by other bacterial toxins.

Moreover, it is possible that an additional host or bacterial factor (filamin, TRIP6 or IQGAP1 are potential candidates based on identified interactions with SseI) is required to convert SseI into a fully active conformation, even in the presence of its substrate(s), as has been shown for other bacterial toxins that contain cysteine protease domains. This additional variable further complicates the definitive identification of the biochemical function(s) of SseI.

## Supplementary Material

PDB reference: C-terminal domain of SseI, native, 4g2b

PDB reference: SeMet derivative, 4g29

Supporting information file. DOI: 10.1107/S0907444912039042/dz5262sup1.pdf

## Figures and Tables

**Table 1:** Crystallographic statistics Values in parentheses are for the highest resolution shell.

Data set	SeMet SAD	Native
Data collection		
Source	NSLS X29A	NSLS X3A
Space group	*P*4_2_2_1_2	*P*2_1_2_1_2_1_
Unit-cell parameters ()		
*a*	52.26	54.05
*b*	52.26	63.19
*c*	132.15	110.62
Wavelength ()	0.9790	1.0809
Resolution ()	50.01.70	50.02.05
No. of reflections	545436	105526
No. of unique reflections	38193	44828
*R*_merge_ † (%)	2.7 (55.9)	2.9 (51.8)
*I*/(*I*)	88.7 (2.1)	45.9 (2.1)
Completeness (%)	99.5 (95.0)	99.5 (98.9)
Multiplicity	15.2 (8.1)	4.4 (3.7)
Refinement		
Resolution ()	19.081.70	27.182.05
No. of reflections	19151	22722
*R*_work_/*R*_free_ ‡ (%)	18.3/21.4	19.4/23.8
No. of atoms		
Total	1472	2877
Protein	1335	2719
Water	137	158
*B* factors (^2^)		
Average	27.2	40.6
Protein	27.4	40.2
Solvent	24.7	48.0
R.m.s. deviations§		
Bond lengths ()	0.019	0.022
Bond angles ()	1.65	1.80
Bond *B* factor (^2^)	1.79	2.00
Ramachandran plot (%)		
Favored regions	91.1	89.3
Allowed regions	8.9	10.7
Outliers	0	0

† *R*_merge_ = 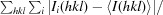 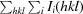 for the intensity *I* of *i* observations of reflection *hkl*.

‡ *R* = 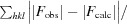 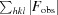, where *F*_obs_ and *F*_calc_ are the observed and model structure factors, respectively; *R*_free_ was calculated using 5% of data that were omitted from refinement.

§ Bond and angle deviations are from ideal values; *B*-factor deviations are between bonded atoms.

## Footnotes

1 Supplementary material has been deposited in the IUCr electronic archive (Reference: DZ5262). Services for accessing this material are described at the back of the journal.
