# Supplementary material for: Structure of the catalytic domain of the Salmonella virulence factor SseI
Source: Acta Crystallogr D Biol Crystallogr. 2012 Nov 9;68(Pt 12):1613–21. doi: 10.1107/S0907444912039042 (PMC3498931; doi:10.1107/S0907444912039042)
Supplement: Supplementary file 1 [file d-68-01613-sup1.pdf]

Supplementary Materials

Material & Methods

Ammonia release assay

Ammonia released by modification of Gln residues was detected using a kit from Sigma. Ammonia reacts with α-ketoglutaric acid (3.4mM) and NADPH (0.23mM) in the presence of L-glutamate dehydrogenase (~12U) to form L-glutamate and oxidized $NADP^+$, which can be measured by the decrease in absorbance at 340nm. In the cuvette version of the assay, 100µL of sample was added to 1mL of test solution while in a modified microplate version, 25µL of sample was added to 250µL of test solution.

Deamidase/Transglutaminase assay

For Gln deamidation, 30mM Z-Gln-Gly (Sigma) in 200mM HEPES pH 7.2, 10mM $CaCl_2$, 10mM DTT was mixed with 9µM enzyme and incubated at 37°C (Yamaguchi *et al.*, 2001). 100µL aliquots were taken out at certain intervals and added to 20µL of 80% TCA to stop the reaction. These samples were then centrifuged to remove precipitated protein and the supernatant tested in the ammonia assay described previously. Both full length SseI (36µg/mL) and 137-322 (18µg/mL) were tested.

For transglutamination, 100mM hydroxylamine was also present in the reaction mix and ammonia release detected as above (Yamaguchi *et al.*, 2001). Additional 100µL aliquots at each time point were also taken out and added to 100µL of stop solution (0.37M $FeCl_3$, 0.67M HCl, 20% TCA), which detects the presence of hydroxamate formation between Gln and NH_2OH.

Samples were centrifuged and the absorbance of the supernatant at 525nm read. Pig liver transglutaminase (Sigma) at 3μM was used as a control in all the assays.

N,N-dimethyl casein (Sigma) (that was methylated at its lysines to prevent cross-linking by and protein precipitaion during transglutamination) was used as a further substrate for deamidation and transglutamination. 1mM casein in 20mM Tris, pH 8.0, 50mM NaCl was incubated with 9μM enzyme at 37°C. Aliquots were taken out and tested for ammonia release as before. The hydroxamate assay could not be used with casein as a substrate since the stop solution contains TCA, which precipitates protein, and in this case would also precipitate any modified casein, unlike the small-molecule substrate used previously.

Protease assay

Two separate assays from Invitrogen were used to check for protease activity. The EnzChek Peptidase/Protease Kit (E33758) uses a mixture of small peptides that are labeled at one terminus with a fluorophore and a quencher at the other. Cleavage results in separation of the fluorophore from the quencher and an increase in fluorescence (excitation 502nm, emission 528nm). Samples were tested at concentrations between 0.04 and 4 mg/mL and were added in a 50μL volume to 50μL of substrate in 10mM Tris pH 7.8. The EnzChek Protease Kit (E6638) uses casein modified with the green dye BODIPY FL. Cleavage results in the release of highly fluorescent peptides (excitation 505nm, emission 598nm). Samples were tested at concentrations between 0.12 and 12 mg/mL and were added in a 50μL volume to 50μL of substrate in 10mM sodium bicarbonate pH 7.8. Papain (Sigma) was used as a positive control with both assays. The effects of added 10mM DTT and 0.2mM E-64 (a cysteine protease inhibitor) (Sigma) were also tested.

N-acetyl transferase assay

The acetlyation of arylamines (Brooke *et al.*, 2003) results in hydrolysis of Acetyl Coenzyme A to give free CoA which has an exposed sulfhydryl group. This –SH group can be detected by 5,5”-dithio-bis(2-nitrobenzoic) acid (DTNB). 10µL of enzyme was added to 90µL of 20mM Tris pH 8.0, 400µM AcCoA (Sigma) and 500µM arylamine substrate at 37°C. After 1 hr, 25µL of 6.4M Guanidine HCl, 100mM HEPES pH 7.2, 5mM DTNB (Sigma) was added to the reaction and the absorbance at 405 nm read. Four different arylamine substrates were tested: Sulfamethazine, 4-aminobenzoic acid, 4-anisidine, and Isoniazid. Both FL (4µg) and C-terminal SseI (2µg) were tested and human cytosolic NAT2 (Sigma) was used as a positive control. Note that the enzymes had to be transferred into buffer without DTT using a PD10 column before use in the assay.

Another way of following NAT activity is to look for the disappearance of free-amines as the substrate is acetylated (Brooke *et al.*, 2003). Reactions were set up as above but the reaction mix also contained 10mM LiK Acetyl phosphate (Sigma) and 32U phosphotransacetylase (Sigma). The LiK acetyl phosphate and phosphotransacetylase recycle AcCoA in order to drive the reaction forward. 100µL of 20% TCA was added to the samples after 1 hr to stop the reaction instead of DTNB. They were then centrifuged and 100µL of the supernatant added to 200µL of 5%(w/v) dimethylaminobenzaldehyde (DMAB). The absorbance at 450nm was read.

Supplementary Figures

Figure Legends:

S1: Domain Structure of SseI. A) Solubility of expressed constructs (yields are in mg of His-tagged protein eluted per L of *E.coli* grown). Limited proteolysis of: B) SseI 1-322, C) SseI 1-138, D) 137-322. Protein samples with increasing ratios of subtilisin were incubated at RT for 30 mins and stable fragments analyzed by N-terminal Edman sequencing. C-terminal positions were estimated based on the molecular weight of the fragment. E) Hypothesized domain structure of SseI based on solubility and limited proteolysis results.

S2: Overall structure of the native catalytic domain dimer, SseI 138-322.

A) Overall fold shown as a cartoon diagram. Helices are shown in blue and beta strands in red. The catalytic triad Cys178, His216, Asp231, and the disulphide-forming Cys258 are shown in orange (sticks). B) Secondary structure alignment with the sequence of chain A of the dimer. Key: helices (H), strands (A), beta turn (β), gamma turn (γ), beta hairpin (⊃). C) Topology of the catalytic domain. Generated by PDBSum (Laskowski, 2009).

S3: Structural matches from the DALI server.

Z-scores above 2 are considered significant (Holm *et al.*, 2008).

S4: Biochemical functions of structural homologs.

Reaction mechanisms for the acyl hydrolase and acyl transferase activities exhibited by structural homologs of SseI's catalytic domain.

S5: Glu Deamidase Assay.

A) Ammonia release from 30 mM Z-Gln-Gly. 9 μM of FL or C-terminal SseI was used, with 3 μM pig liver transglutaminase (+100 mM NH_2OH) as a control. B) Ammonia release from 1 mM casein. 9 μM of FL or C-terminal SseI was used, with 3 μM pig liver transglutaminase (+100 mM NH_2OH) as a control.

S6: Tranglutaminase Assay.

A) Ammonia release from 30 mM Z-Gln-Gly + 100 mM hydroxylamine. 9 μM of FL or C-terminal SseI was used, with 3 μM pig liver TG as a control. B) Ammonia release from 1 mM casein + 100 mM hydroxylamine. 9 μM of FL or C-terminal SseI was used, with 3 μM pig liver TG as a control. C) Hydroxamate formation from 30 mM Z-Gln-Gly + 100 mM hydroxylamine. 9 μM of FL or C-terminal SseI was used, with 3 μM pig liver TG as a control.

S7: Protease Assay.

A) Protease assay using fluorescent peptide. 0.2-2.0 mg/mL of FL and C-terminal SseI were used, with papain as a control. Addition of 10 mM DTT did not alter the activity of SseI. Additon of 0.2 mM E-64 completely ablated papain activity. B) Protease assay using fluorescent casein. 0.06-6.0 mg/mL of FL and C-terminal SseI were used, with papain as a control. Addition of 10 mM DTT did not alter the activity of SseI. Additon of 0.2 mM E-64 completely ablated papain activity.

S8: N-acetyl transferase Assay.

A) Sulfhydryl release from 400 μM AcetylCoA and 500 μM substrate. Concentrations used

were: SseI FL (40 μg/mL), C-terminal (20 μg/mL), and NAT2 (20 μg/mL). B) Amine disappearance measured in 400 μM AcetylCoA and 500 μM substrate. Concentrations used were: SseI FL (40 μg/mL), C-terminal (20 μg/mL), and NAT2 (20 μg/mL).

S1:

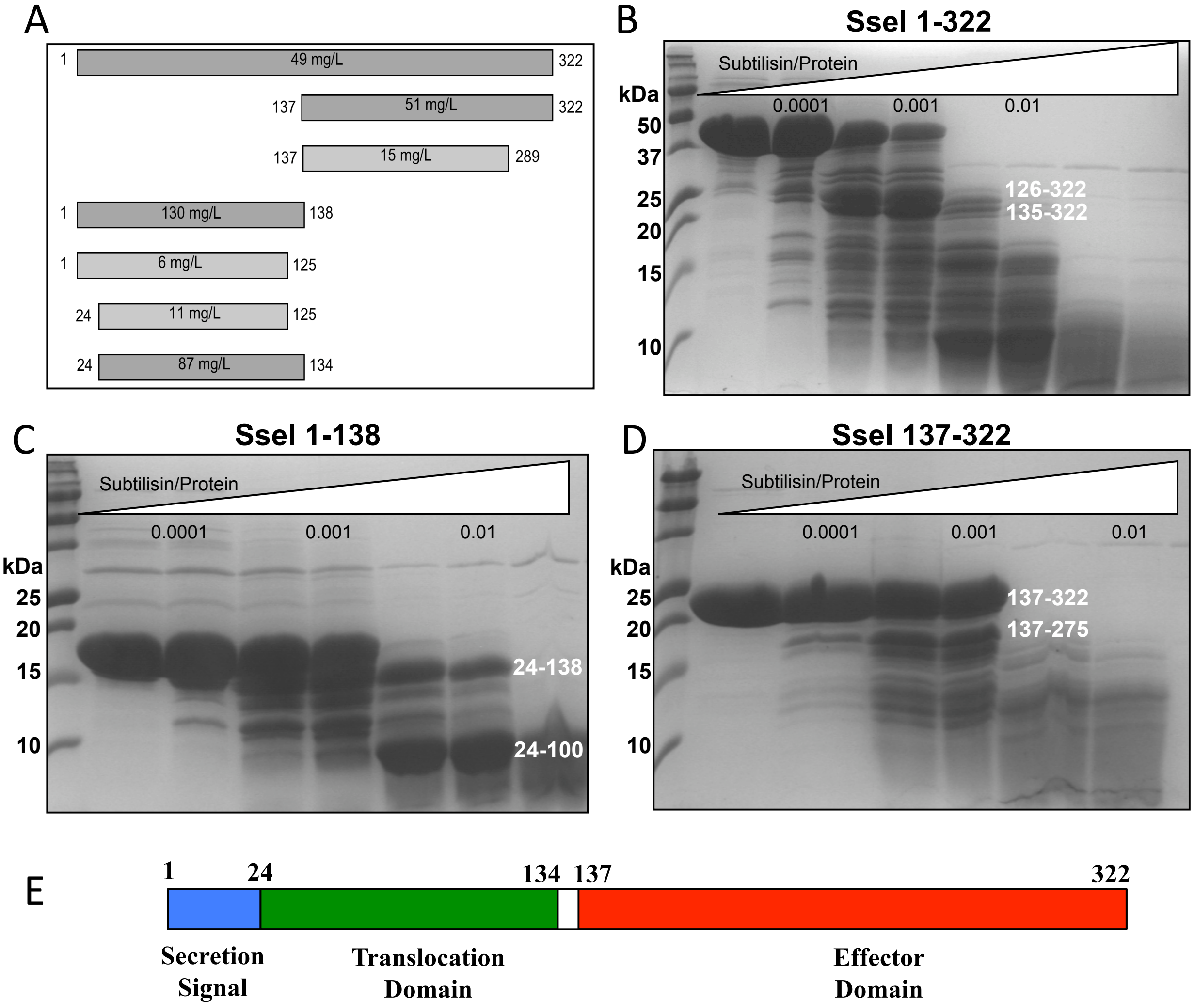
A
1
49 mg/L
322
137
51 mg/L
322
137
15 mg/L
289
1
130 mg/L
138
1
6 mg/L
125
24
11 mg/L
125
24
87 mg/L
134
B
SseI 1-322
Subtilisin/Protein
kDa
0.0001
0.001
0.01
50
37
25
20
15
10
126-322
135-322
C
SseI 1-138
Subtilisin/Protein
0.0001
0.001
0.01
kDa
25
20
15
10
24-138
24-100
D
SseI 137-322
Subtilisin/Protein
0.0001
0.001
0.01
kDa
25
20
15
10
137-322
137-275
E
1
24
134
137
322
Secretion Signal
Translocation Domain
Effector Domain

S2:

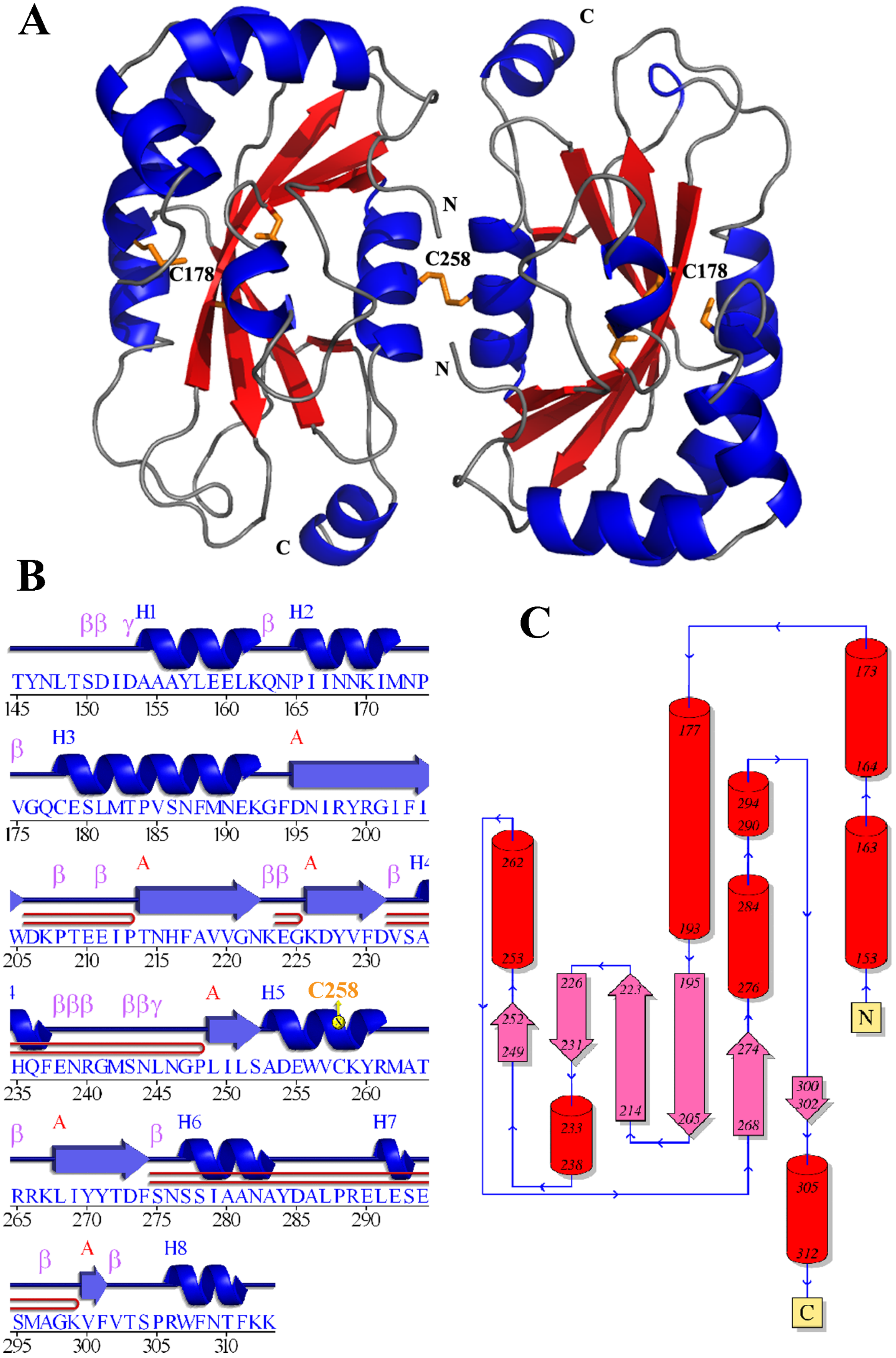
A
C
N
C258
C178
C178
N
C
B
H1
H2
TYNLTSDIDAAAYLEELKQNPIINNKIMNP
145 150 155 160 165 170
H3
VGQCESLMTPVSNFMNEKGFDNIRYRGIFI
175 180 185 190 195 200
WDKPTEEIPTNHFAVVGNKEGKDYVFDVSA
205 210 215 220 225 230
H5
C258
HQFENRGMSNLNGPLILSADEWVCKYRMAT
235 240 245 250 255 260
H6
H7
RRKLIYYTDFSNSSIAANAYDALPRELESE
265 270 275 280 285 290
H8
SMAGKVFVTSPRWFNTFKK
295 300 305 310
C
N
C

S3:

PDB ID	Z-Score	rmsd	No. of residues	Length of alignment	% sequence similarity	Description
2ebf	7.5	3.3	711	126	20	Toxin from *Pasteurella multicoda*
3c9q	6.6	3.6	195	111	9	Uncharacterized human C8ORF32
2z8t	6.5	3.4	185	109	15	Protein-glutaminase, *Cryseobacterium ploteolyticum*
2bsz	6.1	3.8	267	117	11	Bacterial arylamine N-acetyltransferase
1e2t	6.0	3.6	274	107	11	Bacterial arylamine N-acetyltransferase
1ukf	5.1	2.8	188	86	9	AvrPpHB, *Pseudomonas syringae* TTSS effector - protease
1gx3	5.1	3.5	276	111	11	Bacterial arylamine N-acetyltransferase
2vfb	5.0	3.7	272	112	11	Bacterial arylamine N-acetyltransferase
1x3w	4.8	3.1	320	98	19	Yeast Peptide N-Glycase
1w4t	4.8	3.6	276	108	7	Bacterial arylamine N-acetyltransferase
2pqt	4.7	3.7	295	116	13	Bacterial arylamine N-acetyltransferase
2pfr	4.6	3.6	290	108	14	Bacterial arylamine N-acetyltransferase
2hly	4.6	3.5	205	99	9	Uncharacterized *Agrobacterium tumefaciens* Atu2299
1g0d	4.6	4.1	666	108	9	Fish transglutaminase
3d9w	4.3	3.6	284	112	9	Bacterial arylamine N-acetyltransferase
2f4m	4.1	3.3	295	97	16	Mouse Peptide N-Glycase
1fl3	4.1	4.0	721	105	10	Mammalian Transglutaminase
2qsg	4.0	3.7	502	108	9	Tranglutaminase-like domain in Rad4, Yeast DNA repair protein
2iho	4.0	3.4	292	89	4	Mushroom lectin domain, *Marasmius oreades*
1l9m	3.8	4.4	681	106	13	Mammalian Transglutaminase
2q3z	3.2	3.7	655	99	9	Mammalian Transglutaminase
1kv3	3.1	4.1	651	94	11	Mammalian Transglutaminase
1cv8	3.0	2.9	173	82	7	Staphopain, protease, *Staphylococcus aureus*

S4:

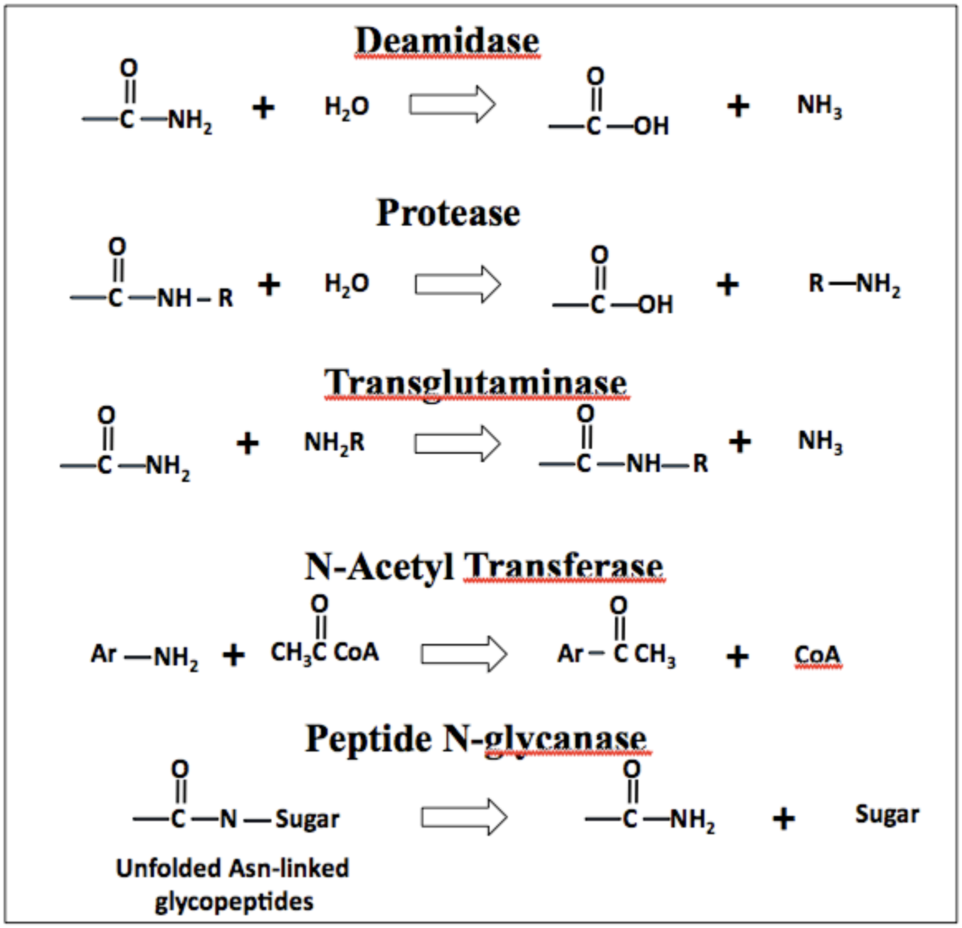
Deamidase
H2O
NH3
Protease
NH – R
R—NH2
Transglutaminase
NH2R
N-Acetyl Transferase
Ar —NH2
CH3C CoA
Ar – C CH3
CoA
Peptide N-glycanase
N—Sugar
Sugar
Unfolded Asn-linked glycopeptides

S5:

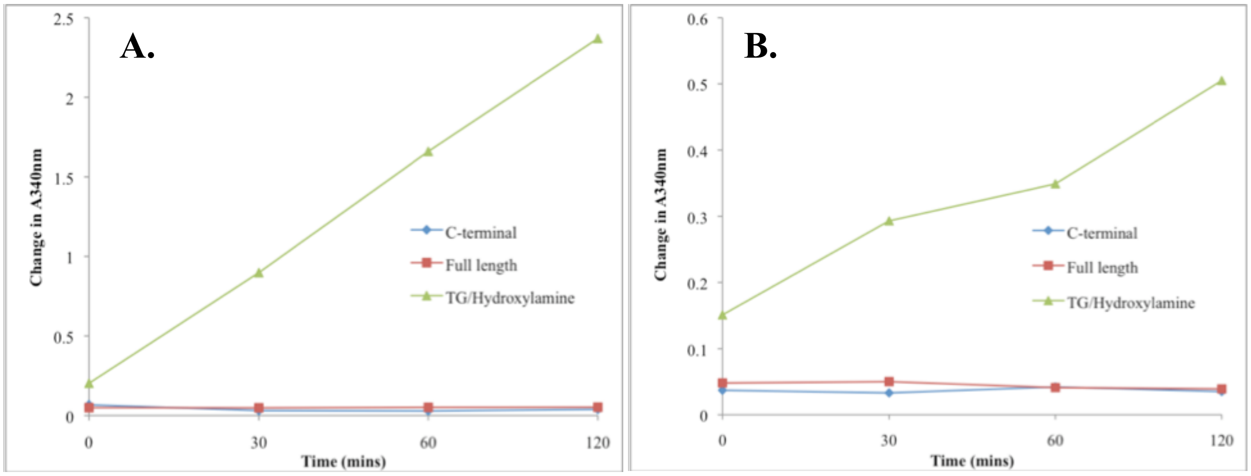
A.
Change in A340nm
Time (mins)
C-terminal
Full length
TG/Hydroxylamine
B.
Change in A340nm
Time (mins)
C-terminal
Full length
TG/Hydroxylamine

S6:

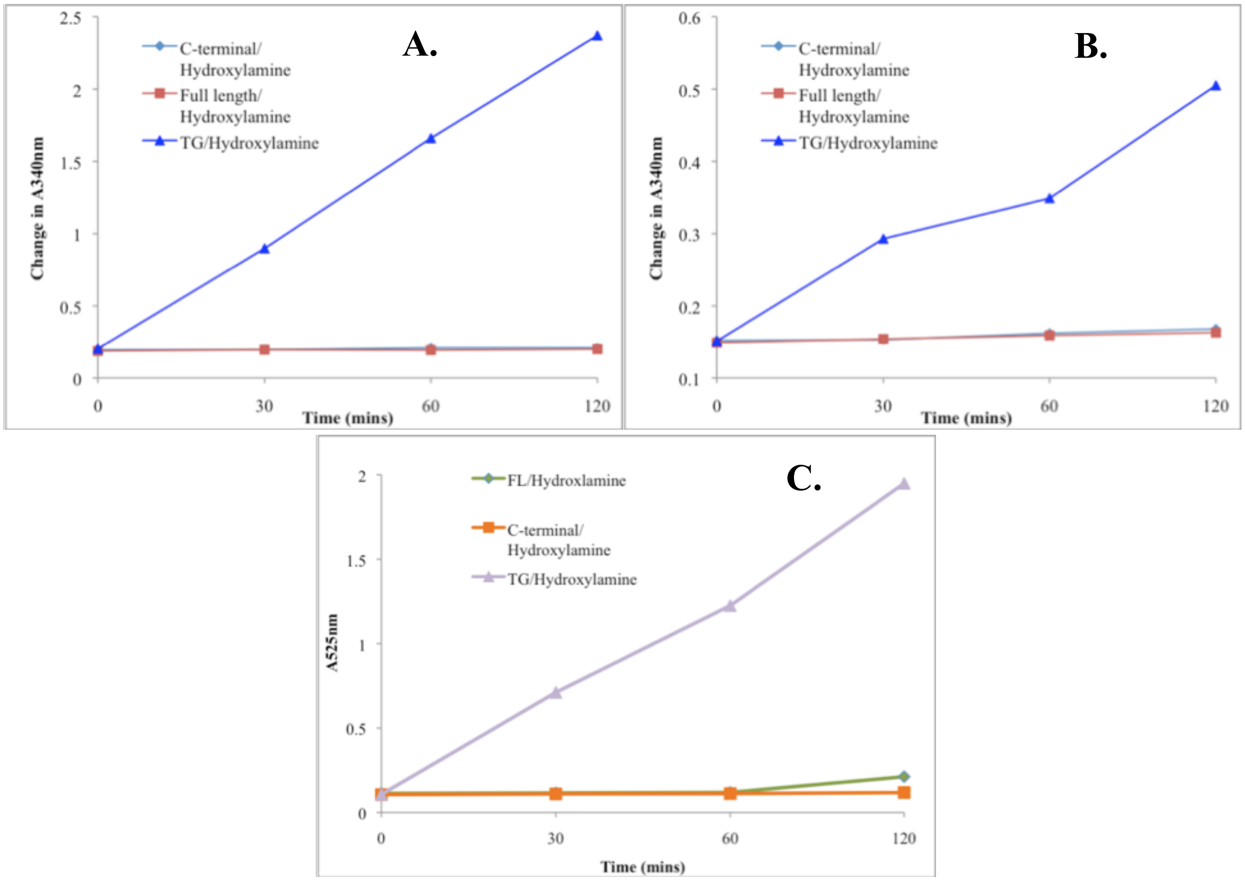
A.
Change in A340nm
Time (mins)
C-terminal/ Hydroxylamine
Full length/ Hydroxylamine
TG/Hydroxylamine
B.
Change in A340nm
Time (mins)
C-terminal/ Hydroxylamine
Full length/ Hydroxylamine
TG/Hydroxylamine
C.
A525nm
Time (mins)
FL/Hydroxlamine
C-terminal/ Hydroxylamine
TG/Hydroxylamine

S7:

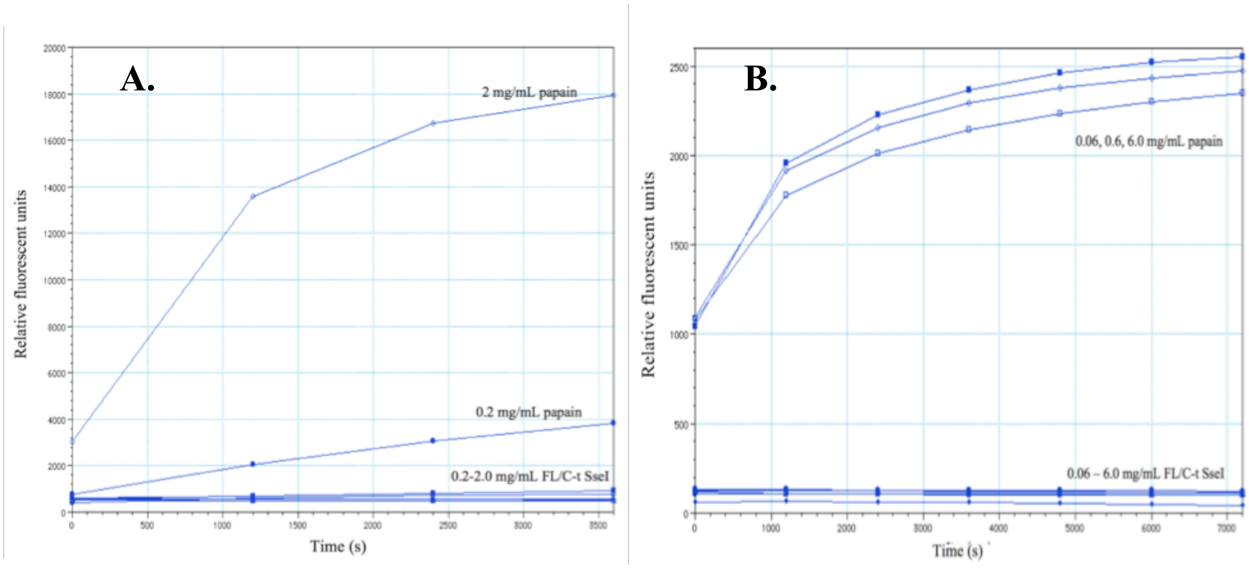
A.
2 mg/mL papain
0.2 mg/mL papain
0.2-2.0 mg/mL FL/C-t SseI
Relative fluorescent units
Time (s)
B.
0.06, 0.6, 6.0 mg/mL papain
0.06 – 6.0 mg/mL FL/C-t SseI
Relative fluorescent units
Time (s)

S8:

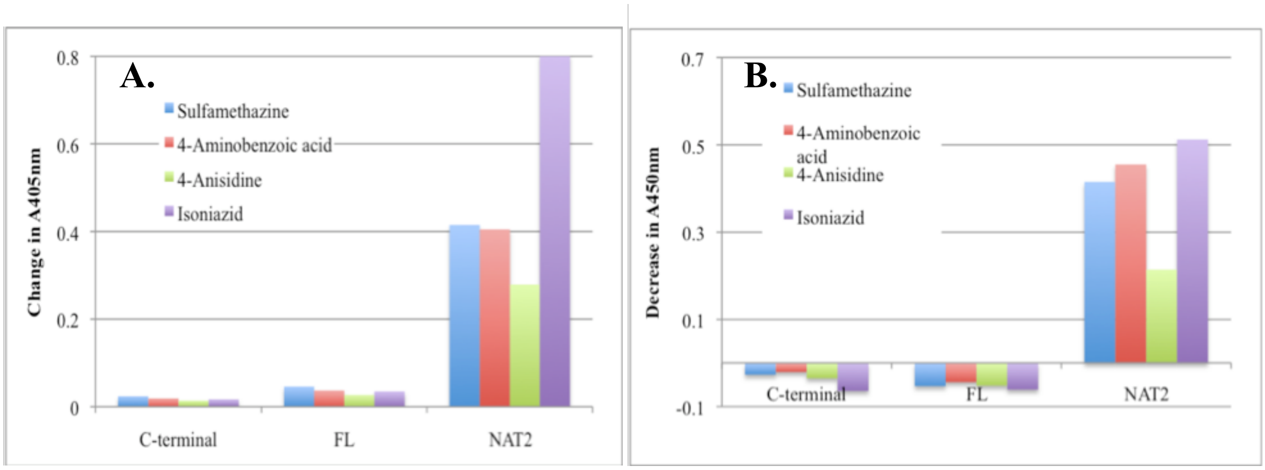
A.
Sulfamethazine
4-Aminobenzoic acid
4-Anisidine
Isoniazid
Change in A405nm
C-terminal
FL
NAT2
B.
Sulfamethazine
4-Aminobenzoic acid
4-Anisidine
Isoniazid
Decrease in A450nm
C-terminal
FL
NAT2

References:

Brooke, E. W., Davies, S. G., Mulvaney, A. W., Pompeo, F., Sim, E. & Vickers, R. J. (2003). *Bioorg Med Chem* **11**, 1227-1234.

Holm, L., Kaariainen, S., Rosenstrom, P. & Schenkel, A. (2008). *Bioinformatics* **24**, 2780-2781.

Laskowski, R. A. (2009). *Nucleic Acids Res* **37**, D355-359.

Yamaguchi, S., Jeenes, D. J. & Archer, D. B. (2001). *Eur J Biochem* **268**, 1410-1421.
